# Beyond Symptom and Category: The Structural Interview as a Clinical and Educational Tool

**DOI:** 10.1192/j.eurpsy.2026.11911

**Published:** 2026-07-31

**Authors:** A. Rebollo Díez, S. Núñez Sevillano

**Affiliations:** Psiquiatría, Complejo Universitario de León, León, Spain

## Abstract

**Introduction:**

The Structural Interview, developed by Otto Kernberg (1981), is a clinical tool designed to dimensionally assess a patient’s psychic functioning. Grounded in Kernberg’s structural model, it allows the exploration of domains such as identity, defense mechanisms, object relations, aggression, moral values, and reality testing (RADIOS). Its application supports the formulation of diagnostic hypotheses from the very first clinical encounter, integrating symptoms within a structural understanding of the subject and their psyche. Moreover, its sequential format facilitates the systematic organization of information, making it a particularly valuable resource in educational contexts.

**Objectives:**

To review the clinical and educational value of the Structural Interview in Psychiatry.

**Methods:**

A narrative review of the specialized literature was conducted regarding the Structural Interview as both a diagnostic and educational tool in Psychiatry. The analysis focused on its clinical applicability and pedagogical value in resident training.

**Results:**

The Structural Interview combines classical psychopathological exploration with a dynamic assessment of psychic functioning. It aims to guide diagnosis by analyzing symptoms and conflicts manifested both in verbal content and in transference phenomena that emerge during the interview.

The model follows a sequential structure: it typically begins with open-ended questions to assess narrative coherence, insight, and expectations, followed by exploration of areas such as interpersonal relationships, intimacy, occupational or academic performance, creativity, and relevant aspects of psychobiographical history such as traumatic events, antisocial behaviors, or significant attachments. The interview systematically examines the six domains of psychic functioning included in the RADIOS acronym to provide an integrated picture of the patient’s clinical presentation and overall functioning.

Techniques such as clarification, confrontation, and interpretation are employed to deepen the discourse, thereby facilitating the understanding of symptoms, conflicts, and defense mechanisms.

From an educational standpoint, the Structural Interview promotes organized, detailed, and dimensional exploration. It provides the evaluator with a structure that transcends symptomatic description and captures the patient’s global functioning, offering diagnostic orientation, prognostic assessment, and a framework for treatment planning.

**Conclusions:**

The Structural Interview facilitates a dimensional understanding of psychic functioning that complements categorical diagnosis. Beyond its clinical value, it fosters the physician–patient alliance and enhances therapeutic planning. Its integration into psychiatric training programs promotes exploration “beyond the symptom” and supports a clinical practice that is rigorous, ethical, and centered on the individual.

**Disclosure of Interest:**

None Declared

